# Prevalence and Related Factors of Depression, Anxiety, Acute Stress, and Insomnia Symptoms Among Medical Staffs Experiencing the Second Wave of COVID-19 Pandemic in Xinjiang, China

**DOI:** 10.3389/fpubh.2021.671400

**Published:** 2021-05-17

**Authors:** Yongzhi Zhao, Junlong Guo, Shuai Liu, Muyeseer Aizezi, Qiong Zeng, Ashenggu Sidike, Raziya Abliz, Aisikaerjiang Kudireti, Yan Xie, Atikan Taineikuli, Bin Zhang

**Affiliations:** ^1^Department of Psychiatry, Nanfang Hospital, Southern Medical University, Guangzhou, China; ^2^First People's Hospital of Kashi, Xinjiang, China; ^3^Guangdong San-jiu Brain Hospital, Guangzhou, China

**Keywords:** COVID-19, mental health, medical staff, resurgence, pandemic

## Abstract

The prevalence and related factors of mental health impact among medical staffs who experienced the second wave of the COVID-19 pandemic in China is unknown. Therefore, this survey was conducted to investigate the prevalence and related factors of depressive, anxiety, acute stress, and insomnia symptoms in medical staffs in Kashi, Xinjiang, China during the second wave of the COVID-19 pandemic. A cross-sectional online survey was conducted among medical staffs working in First People's Hospital of Kashi, Xinjiang. The questionnaire collected demographic data and self-design questions related to the COVID-19 pandemic. The Impact of Events Scale-6, the Insomnia Severity Index, the Patient Health Questionnaire-9, the Generalized Anxiety Disorder Scale-7, the Perceived Social Support Scale, the Chinese Big Five Personality Inventory-15, and the Trait Coping Style Questionnaire were used to measure psychological symptoms or characteristics. Binary logistic regression was carried out to examine the associations between socio-demographic factors and symptoms of depression, anxiety, stress, and insomnia. In total, data from 123 participants were finally included, among which the prevalence rate of depressive, anxiety, acute stress, and insomnia symptoms is 60.2, 49.6, 43.1, and 41.1%, respectively. The regression model revealed that minority ethnicity, being worried about infection, spending more time on following pandemic information, and neurotic personality were positively associated with the mental health symptoms, while extraversion personality, higher education level, and better social support were negatively associated. In our study, the prevalence of mental health impact was high among medical staffs in Kashi, China who experienced the second wave of the COVID-19 pandemic. Several factors were found to be associated with mental health conditions. These findings could help identify medical staffs at risk for mental health problems and be helpful for making precise mental health intervention policies during the resurgence. Our study may pave way for more research into Xinjiang during the COVID-19 pandemic.

## Introduction

The coronavirus disease 2019 (COVID-19), which first broke out in Wuhan, China at the end of 2019 brought about a global public health emergency. On January 23rd, 2020, the first two confirmed cases were reported in Xinjiang, China, and Xinjiang was going through a major public health event (1). Under the effective preventive measures of the Chinese government, the pandemic has gradually been brought under control in Xinjiang, as there has been neither a new confirmed nor new asymptomatic indigenous case in Xinjiang since August 18th, 2020 (2). Furthermore, since August 21st, 2020, neither new confirmed nor new asymptomatic indigenous cases had been reported in the mainland of China (3). The situation had lasted for about 2 months, which was much longer than the 2-week incubation of COVID-19. Therefore, it can be stated that the first wave of the pandemic was ended in the mainland of China. However, the pandemic began to rebound in the mainland of China, starting with Xinjiang where a new indigenous asymptomatic case was reported again on October 24th (4). Since then, there has been a resurgence of the pandemic first in Xinjiang followed by other provinces of China, putting the whole country into another round of major public health event.

Major public health events, such as the outbreak of COVID-19, generate great concern as well as mental health problems among people, especially among medical staffs. A meta-analysis revealed that the COVID-19 pandemic increases the mental health problems of the global population, particularly health care workers (5). Some other studies conducted during the outbreak period also showed the high prevalence of mental health problems such as depression, anxiety, insomnia, and acute stress among medical staffs (6–9).

Several factors are found to be associated with these mental health problems. Medical workers who have direct clinical contact with infected patients, are suspected cases, or work in the worst affected area are found more likely to experience anxiety symptoms (6). Zhang's study suggests that insomnia symptoms are positively associated with low education level, currently working in an isolation unit, worried about being infected, perceiving lack of helpfulness in terms of psychological support from the news or social media concerning COVID-19, and having very strong uncertainty regarding effective disease control (7). Besides, acute stress disorder is found to be associated with psychosomatic symptoms as well as hostility (9). Furthermore, social support, coping style, and personality are also considered to play an important role in the prevalence of mental health problems. A study done during the early outbreak of COVID-19 identifies that levels of social support for medical staffs are significantly associated with self-efficacy and sleep quality and negatively associated with the degree of anxiety and stress (10). Another study reveals that the positive coping mechanism was negatively correlated with anxiety (8). As for personality, extraversion, agreeableness, conscientiousness, and openness are found negatively correlated with generalized anxiety and depressive symptoms while neuroticism is positively correlated (11).

Although several studies have analyzed the mental health status of medical staffs during major public health events, only a few studies were done in Xinjiang, China (12). Besides, most of the studies were conducted during the outbreak period of COVID-19 rather than the resurgence period, leaving the health status of medical staffs during such a special major public health event still unknown. Moreover, as Xinjiang was the first place where the second wave of the pandemic broke out, the investigation and study done here would be more meaningful and representative. In view of this, we did this survey to investigate depressive, anxiety, acute stress, and insomnia symptoms to explore the related socio-psychological factors among medical staffs who experienced the resurgence of the COVID-19 pandemic in Kashi, China.

## Methods

### Study Design and Samples

The cross-sectional survey was conducted online among medical staffs working in the First People's Hospital of Kashi, Xinjiang, China. It was started on November 5th, 2020 and ended on November 12th, 2020, when Kashi was experiencing the second round of the COVID-19 pandemic. Participants who met the following criteria were included: (1) medical staff, (2) could read a Chinese questionnaire, (3) WeChat user, and (4) volunteered for the survey. The exclusion criterion was being unable to understand the questionnaire. Our investigators forwarded the questionnaire to different WeChat groups of medical staffs to recruit participants. Before the survey got started, the purpose and significance of the survey were introduced briefly to all participants, and participants' consent was necessary for further continuation of the survey. Before the final submission, participants could proceed only if all questions in the survey were answered. The data were recorded automatically. People who completed the questionnaire were encouraged to forward the survey to others. This study was approved by the Ethics Committee of Nanfang Hospital, Southern Medical University. To guarantee the participants' privacy, the survey was conducted anonymously.

### Measures

Demographic data were collected at the beginning of the survey. Self-designed questions related to the COVID-19 pandemic, such as infection status and contact with feverish or infected patients, were also recorded.

The Patient Health Questionnaire-9 (PHQ-9) was included to assess depressive symptoms (13), of which the total scores can be categorized into normal (0–4), mild (5–9), moderate (10–14), and severe (15–27) depression. The cutoff score of 5 for PHQ-9 was adopted in this study.

The Generalized Anxiety Disorder Scale-7 (GAD-7) (14) was used to detect anxiety symptoms, with the categorization of the total score into normal (0–4), mild (5–9), moderate (10–14), and severe (15–21) anxiety. The cutoff score was set as 5 for GAD-7.

The Impact of Event Scale-6 (IES-6) (15) was used to identify acute stress symptoms. The average score “S” of IES-6 is categorized as follows: S < 1.09 = normal: 1.09 ≤ S < 1.5 = showing stress symptoms; S ≥ 1.5 = may be diagnosed with PTSD (16). The cut-off score of 7 (the average score S ≥1.09) was applied in this study.

As for the evaluation of insomnia symptoms, the Insomnia Severity Index (ISI) was administered (17), of which the classifications of the total score were categorized into normal (0–7), mild (8–14), moderate (15–21), and severe (22–28) insomnia. A total score of ≥8 is considered to be having symptoms of insomnia.

The Chinese Big Five Personality Inventory-15 (CBF-PI-15), which consists of five independent factors including extraversion (E), agreeableness (A), conscientiousness (C), neuroticism (N; emotional stability), and openness to experience (O), was used to measure personality in this study (18). Scoring higher in each facet implies its positive tendency in that dimension of personality.

The Trait Coping Style Questionnaire (TCSQ) was used to assess our subjects' coping style (19). TCSQ consists of two sub-scales, of which the higher score in each sub-scale reflects the higher tendency of positive or negative coping style.

The Perceived Social Support Scale (PSSS) was performed to analyze social support (20). The total score ranges from 12 to 84, which can be categorized into 3 different levels: poor (12–36), moderate (37–60), and strong (61–84) social support.

### Statistical Analysis

The categorical variables in this study were presented with frequency and percentage while the continuous variables were reported with mean and standard deviation. In univariate analyses, a Chi-square test or *t*-test was used to examine the associations of demographics, pandemic-related, and psychological factors with depressive, anxiety, insomnia, and acute stress symptoms. The factors showed significance in the univariate analyses were included in further binary logistic regression analyses. The regression model was adjusted for gender and age by using the enter method while other demographic, pandemic-related, and psychological factors were analyzed by using the forward likelihood ratio method. All analyses were two-tailed with an alpha level set at *P* < 0.05 and were conducted using SPSS software 22.0.

## Results

### Demographic Characteristics

Data from 123 participants were obtained and none were excluded prior to data analysis. The average time the participants spent finishing the questionnaire was about 17 min. Of the total sample, 34 participants (27.6%) were male, and the mean (SD) age was 36.98 (7.88) years. Most of the participants had a bachelor's degree or below (*n* = 99, 80.5%) and were non-smokers (*n* = 102, 82.9%). While 71 participants (57.7%) were doctors, 37 (30.1%) were nurses, and 15 (12.2%) were other medical staffs. Of the total number of participants, 35 (28.5%) participants had a sub-senior or senior title, 53 (43.1%) consumed alcohol, 19 (15.4%) worked in fever outpatient/ emergency/ isolation unit/ intensive care unit, and 7 (5.7%) had infected people around them. None of the participants ever got infected with COVID-19 (shown in Table 1).

**Table 1:** Demographic characteristic of the total sample (*N* = 123).

**Factors**	**Frequency (%)**
**Gender**	
Male	34(27.6)
Female	89(72.4)
**Age (Mean** **±** **SD)**	36.98 ± 7.88
**Ethnicity**	
Minority	61(49.6)
Han	62(50.4)
**Education level**	
Bachelor or below	99(80.5)
Master or doctorate	24(19.5)
**Staff type**	
Doctor	71(57.7)
Nurse	37(30.1)
Others	15(12.2)
**Staff title**	
None or junior	48(39.0)
Middle	40(32.5)
Sub-senior or senior	35(28.5)
**Working department**	
Fever outpatient/ Emergency/ Isolation unit/ ICU	19(15.4)
Normal outpatient or inpatient unit	78(63.4)
Others (Medical laboratory/ Pharmacy/ Administrative department etc.)	26(21.1)
**Alcohol use**	
No	70(56.9)
Yes	53(43.1)
**Smoking**	
No	102(82.9)
Yes	21(17.1)
**Work requires contact with feverish or infected patients**	
No	108 (87.8)
Yes	15 (12.2)
**Infected with COVID-19**	
No	123(100)
**People around you infected with COVID-19**	
No	116(94.3)
Yes	7(5.7)

*SD, standard deviation; ICU, intensive care unit*.

### Prevalence of Symptoms of Acute Stress, Insomnia, Depression, and Anxiety

The prevalence of the studied mental health symptoms among the total sample was 60.2% for depressive, 49.6% for anxiety, 43.1% for acute stress, and 41.4% for insomnia symptoms, respectively. The prevalence of symptoms of the four mental health conditions was higher among participants who had a bachelor's degree or below, were worried about infection, spent more time on pandemic information, had moderate social support, scored lower in positive coping sub-scale of TCSQ and extraversion sub-scale of CBF-PI-15, and scored higher in neuroticism sub-scale of CBF-PI-15. The symptoms of anxiety, acute stress and insomnia were more prevalent among participants of minority ethnicities. Furthermore, the participants with depressive symptoms were more likely to be female, having none or junior staff title, smoking, and with a high score in the negative coping sub-scale of TCSQ (shown in Tables 2, 3).

**Table 2:** Prevalence of symptoms of acute stress, insomnia, depression, and anxiety stratified by demographic factors.

	**Depressive symptoms**			**Anxiety symptoms**			**Acute Stress symptoms**			**Insomnia symptoms**		
	**No(*n* = 49)**	**Yes(*n* = 74)**	***P***	**No(*n* = 62)**	**Yes(*n* = 61)**	***P***	**No(*n* =70)**	**Yes(*n* = 53)**	***P***	**No(*n* = 61)**	**Yes(*n* = 62)**	***P***
**Total**	49(39.8%)	74(60.2%)		62(50.4%)	61(49.6%)		70(56.9%)	53(43.1%)		61(49.6%)	62(50.4%)	
**Gender**												
Male	21(42.9%)	13(17.6%)	**0.002**	21(30.6%)	13(24.6%)	0.453	22(31.4%)	12(22.6%)	0.281	21(34.4%)	13(21.0%)	0.095
Female	28(57.1%)	61(82.4%)		28(69.4%)	61(75.4%)		48(68.6%)	41(77.4%)		40(65.6%)	49(79.0%)	
**Age (Mean** **±** **SD)**	38.49 ± 8.51	35.97 ± 7.32	0.083	37.18 ± 8.21	36.77 ± 7.59	0.776	36.64 ± 8.10	37.42 ± 7.62	0.592	37.59 ± 7.64	36.37 ± 8.12	0.393
**Ethnicity**												
Minority	20(40.8%)	41(55.4%)	0.113	24(38.7%)	37(60.7%)	**0.015**	29(41.4%)	32(60.4%)	**0.037**	24(39.3%)	37(59.7%)	**0.024**
Han	29(59.2%)	33(44.6%)		38(61.3%)	24(39.3%)		41(58.6%)	21(39.6%)		37(60.7%)	25(40.3%)	
**Education level**												
Bachelor or below	35(71.4%)	64(86.5%)	**0.039**	44(71.0%)	55(90.2%)	**0.007**	52(74.3%)	47(88.7%)	**0.046**	43(70.5%)	56(90.3%)	**0.006**
Master or doctorate	14(28.6%)	10(13.5%)		18(29.0%)	6(9.8%)		18(25.7%)	6(11.3%)		18(29.5%)	6(9.7%)	
**Staff type**												
Doctor	31(63.3%)	40(54.1%)	0.595	35(56.5%)	36(59.0%)	0.951	41(58.6%)	30(56.6%)	0.259	40(65.6%)	31(50.0%)	0.176
Nurse	13(26.5%)	24(32.4%)		19(30.6%)	18(29.5%)		18(25.7%)	19(35.8%)		16(26.2%)	21(33.9%)	
Others	5(10.2%)	10(13.5%)		8(12.9%)	7(11.5%)		11(15.7%)	4(7.5%)		5(8.2%)	10(16.1%)	
**Staff title**												
None or junior	13(26.5%)	35(47.3%)	**0.040**	19(30.6%)	29(47.5%)	0.158	29(41.4%)	19(35.8%)	0.561	18(29.5%)	30(48.4%)	0.091
Middle	17(34.7%)	23(31.1%)		23(37.1%)	17(27.9%)		20(28.6%)	20(37.7%)		22(36.1%)	18(29.0%)	
Sub-senior or senior	19(38.8%)	16(21.6%)		20(32.3%)	15(24.6%)		21(30.0%)	14(26.4%)		21(34.4%)	14(22.6%)	
**Smoking (Yes)**	14(28.6%)	7(9.5%)	**0.006**	11(17.7%)	10(16.4%)	0.842	12(17.1%)	9(17.0%)	0.981	10(16.4%)	11(17.7%)	0.842
**Alcohol use (Yes)**	25(51.0%)	28(37.8%)	0.148	30(48.4%)	23(37.7%)	0.232	32(45.7%)	21(39.6%)	0.499	30(49.2%)	23(37.1%)	0.176
**Working department**												
Fever outpatient/ Emergency/ Isolation unit/ ICU	8(16.3%)	11(14.9%)	0.826	9(14.5%)	10(16.4%)	0.906	10(14.3%)	9(17.0%)	0.359	11(18.0%)	8(12.9%)	0.386
Normal outpatient or inpatient unit	32(65.3%)	46(62.2%)		39(62.9%)	39(63.9%)		48(68.6%)	30(56.6%)		40(65.6%)	38(61.3%)	
Others (Medical laboratory/ Pharmacy/ Administrative department etc.)	9(18.4%)	17(23.0%)		14(22.6%)	12(19.7%)		12(17.1%)	14(26.4%)		10(16.4%)	16(25.8%)	

*ICU, intensive care unit. The bold values are significant P < 0.05*.

**Table 3:** Prevalence of symptoms of acute stress, insomnia, depression, and anxiety stratified by pandemic-related factors and psychological characteristic factors.

	**Depressive symptoms**			**Anxiety symptoms**			**Acute Stress symptoms**			**Insomnia symptoms**		
	**No(*n* = 49)**	**Yes(*n* = 74)**	***P***	**No(*n* = 62)**	**Yes(*n* = 61)**	***P***	**No(*n* = 70)**	**Yes(*n* = 53)**	***P***	**No(*n* = 61)**	**Yes(*n* = 62)**	***P***
**COVID-19-related questions**												
Work requires contact with feverish or infected patients (Yes)	5(10.2%)	10(13.5%)	0.583	7(11.3%)	8(13.1%)	0.757	8(11.4%)	7(13.2%)	0.765	8(13.1%)	7(11.3%)	0.757
Infected with COVID-19 (No)	49(100%)	74(100%)	-	62(100%)	61(100%)	-	70(100%)	53(100%)	-	61(100%)	62(100%)	-
People around you infected with COVID-19 (Yes)	1(2.0%)	6(8.1%)	0.306	4(6.5%)	3(4.9%)	1.000	4(5.7%)	3(5.7%)	1.000	2(3.3%)	5(8.1%)	0.449
Worried about infection (Yes)	25(51.0%)	66(89.2%)	**<0.001**	38(61.3%)	53(86.9%)	**0.001**	43(61.4%)	48(90.6%)	**<0.001**	36(59.0%)	55(88.7%)	**<0.001**
Time spent on pandemic information everyday (>2 h)	18(36.7%)	38(51.4%)	0.111	22(35.5%)	34(55.7%)	**0.024**	29(41.4%).	27(50.9%)	0.294	28(45.9%)	28(45.2%)	0.934
Time spent on pandemic information before sleep (≥30 min)	17(34.7%)	41(55.4%)	**0.024**	24(38.7%)	34(55.7%)	0.059	24(34.3%)	34(64.2%)	**0.001**	22(36.1%)	36(58.1%)	**0.015**
**Psychological characteristics**												
**CBF-PI-15**												
Extraversion	11.31 ± 3.21	9.66 ± 3.10	**0.005**	11.03 ± 3.24	9.59 ± 3.08	**0.013**	11.39 ± 3.04	8.91 ± 2.95	** <0.001**	10.97 ± 3.04	9.68 ± 3.31	**0.026**
Agreeableness	14.35 ± 3.50	14.16 ± 2.65	0.740	14.39 ± 3.27	14.08 ± 2.73	0.576	13.99 ± 3.39	14.57 ± 2.41	0.291	14.23 ± 3.44	14.24 ± 2.54	0.982
Conscientiousness	14.02 ± 3.53	13.59 ± 2.74	0.454	13.89 ± 3.38	13.64 ± 2.74	0.656	13.67 ± 3.50	13.89 ± 2.42	0.688	13.85 ± 3.62	13.68 ± 2.44	0.754
Neuroticism	16.08 ± 2.00	19.23 ± 2.18	**<0.001**	16.44 ± 2.17	19.54 ± 2.03	**<0.001**	16.90 ± 2.40	19.40 ± 2.17	**<0.001**	16.90 ± 2.58	19.03 ± 2.19	**<0.001**
Openness	10.37 ± 4.25	10.22 ± 3.92	0.840	10.23 ± 4.12	10.33 ± 3.99	0.889	10.39 ± 3.98	10.13 ± 4.16	0.732	10.03 ± 4.33	10.52 ± 3.75	0.509
**TCSQ**												
Positive	35.20 ± 6.51	30.59 ± 6.80	**<0.001**	34.90 ± 6.64	29.92 ± 6.57	**<0.001**	34.47 ± 6.86	29.74 ± 6.39	**<0.001**	34.49 ± 7.10	30.40 ± 6.40	**0.001**
Negative	22.10 ± 7.62	25.81 ± 5.94	**0.003**	22.71 ± 7.31	25.98 ± 6.01	**0.008**	23.41 ± 7.55	25.55 ± 5.71	0.077	23.28 ± 7.72	25.37 ± 5.80	0.092
**PSSS**												
Strong(61–84)	36(73.5%)	32(43.2%)	**0.001**	46(74.2%)	22(36.1%)	**<0.001**	48(68.6%)	20(37.7%)	**0.001**	40(65.6%)	28(45.2%)	**0.023**
Moderate or poor(12–60)	13(26.5%)	42(56.8%)		16(25.8%)	39(63.9%)		22(31.4%)	33(62.3%)		21(34.4%)	34(54.8%)	

*PSSS, Perceived Social Support Scale; TCSQ, Trait Coping Style Questionnaire; CBF-PI-15, The Chinese Big Five Personality Inventory-15. The bold values are significant P < 0.05*.

Regarding the prevalence of the four mental health conditions among different types of medical staffs, the prevalence of depressive symptoms among doctors, nurses, and other medical staffs (including medical laboratory/pharmacy/administrative department etc.) was 56.34, 64.86, and 66.67%, respectively. The prevalence of anxiety symptoms was 50.71, 48.65, and 46.67%, respectively. The prevalence of acute stress symptoms was 42.25, 51.35, and 26.67%, respectively. And the prevalence of insomnia symptoms among doctors, nurses, and other medical staffs was 43.66, 56.76, and 66.67%, respectively.

### Factors Associated With Symptoms of Acute Stress, Insomnia, Depression, and Anxiety

The results of binary logistic regression analysis of the related factors of the four mental health conditions are shown in Table 4. Scoring higher in the neuroticism sub-scale of CBF-PI-15 was found to indicate a higher risk of the four mental health symptoms (range, adjusted ORs 1.41–1.91). Compared with those who did not worry about infection, participants that showed their worrying had a higher risk of depressive symptoms (adjusted OR, 3.43; 95%CI, 1.38–2.22). As for anxiety symptoms, participants of minority ethnicities were found to have a higher risk than that of Han ethnicity (adjusted OR, 3.06 95%CI, 1.08–8.65). Meanwhile, anxiety symptoms were more likely among those who had moderate or poor social support when compared with those having strong social support (adjusted OR, 4.68; 95%CI, 1.68–13.03). In addition, participants who spent more than 30 min on pandemic information before sleep were more likely to experience acute stress symptoms (adjusted OR, 3.14; 95%CI 1.25–1.88). On the contrary, a lower risk of acute stress symptoms was associated with a higher score in the extraversion sub-scale of CBF-PI-15 (adjusted OR, 0.78; 95%CI, 0.66–0.91). Moreover, participants with higher education level were also less likely to have insomnia symptoms and anxiety symptoms (range, adjusted ORs 0.19–0.28).

**Table 4:** Logistic regression analysis of factors related to mental health symptoms.

	**Depressive symptoms**			**Anxiety symptoms**			**Acute Stress symptoms**			**Insomnia symptoms**		
	**Adjusted OR**	**95% CI**	***P***	**Adjusted OR**	**95% CI**	***P***	**Adjusted OR**	**95% CI**	***P***	**Adjusted OR**	**95% CI**	***P***
**Gender**												
Male		Ref			Ref			Ref			Ref	
Female	1.84	0.60–5.64	0.286	0.51	0.14–1.86	0.310	1.23	0.43–352	0.697	1.04	0.40–2.68	0.940
**Age**	0.97	0.91–1.03	0.263	1.03	0.97–1.10	0.355	1.04	0.98–1.10	0.222	0.99	0.94–1.05	0.767
**Ethnicity**												
Han		-			Ref			-			-	
Minority		-		3.06	1.08–8.65	**0.035**		-			-	
**Education level**												
Bachelor or below		-			Ref			-			Ref	
Master or doctorate		-		0.19	0.04–0.86	**0.030**		-		0.28	0.09–0.86	**0.026**
**Worried about infection**												
No		Ref			-			-			-	
Yes	3.43	1.12–10.51	**0.031**		-			-			-	
**Time spent on pandemic information before sleep**												
<30 min		-			-			Ref			-	
≥30 min		-			-		3.14	1.25–1.88	**0.015**		-	
**CBF-PI-15**												
Extraversion		-			-		0.78	0.66–0.91	**0.002**		-	
Neuroticism	1.75	1.39–2.22	**<0.001**	1.91	1.48–2.47	**<0.001**	1.53	1.25–1.88	**<0.001**	1.41	1.19–1.67	**<0.001**
**PSSS**												
Strong(61–84)		-			Ref			-			-	
Moderate or poor(12–60)		-		4.68	1.68–13.03	**0.003**		-			-	

*OR, odds ratio; CI, confidence interval; CBF-PI-1, The Chinese Big Five Personality Inventory-15; PSSS, Perceived Social Support Scale*.

*Binary logistic regression controlled for gender and age (enter method) as well as other demographic factors, pandemic-related factors, and psychological factors significantly associated with a certain kind of mental health problem (forward likelihood ratio method). The bold values are significant P < 0.05*.

## Discussion

In this study, a total of 123 participants were investigated, of which approximately 41.4–60.2% exhibited symptoms of depression, anxiety, acute stress, and insomnia. We identified that minority ethnicity, being worried about the pandemic, spending more time on pandemic information, and neurotic personality were positively associated with the four mental health conditions, while extraversion personality, higher education level, and better social support were negatively associated.

The prevalence of the four mental health symptoms studied in the present study is much higher than previous findings. A previous meta-analysis showed that the pooled prevalence of depression and anxiety among health care workers during the COVID-19 pandemic is 22.8 and 23.2%, respectively (21). Another study, which also used ISI and the same cut-off score as this study to detect insomnia symptoms among medical staffs, presented a prevalence of 36.1% for insomnia symptoms (7). Meanwhile, Wang's study found that the prevalence of acute stress disorder symptoms is 38.3% among frontline health professionals, but used a different questionnaire than this study to measure acute stress reaction (9). The higher prevalence of mental health symptoms in our study could be due to the resurgence of the COVID-19 pandemic. While the first round of the pandemic was generally controlled in most parts of mainland China, Xinjiang, especially Kashi City, was undergoing the second round of COVID-19 pandemic in advance. The existence of the COVID-19 pandemic arouses people's fear, worry and uncertainty about infection. Taha's study demonstrates that individuals with a high intolerance of uncertainty are more likely to perceive the pandemic as threatening, predicting elevated levels of anxiety (22). An analysis done by Bakioglu also indicates a positive relationship between fear of COVID-19 and intolerance of uncertainty, depression, anxiety, and stress (23). In addition, a structural equation modeling reveals that intolerance of uncertainty is strongly associated with anxiety sensitivity, in turn influencing both insomnia severity and sleep quality via depression and anxiety (24). From these, we could infer that the resurgence of the COVID-19 pandemic leads to worry, fear, and uncertainty among medical staffs. Although the successful experience of fighting against the first COVID-19 pandemic may also help in dealing with the second wave of the pandemic, the resurgence increases the uncertainty of whether the pandemic could be brought under control or not, resulting in a higher prevalence of mental health conditions. Another reason for the higher prevalence may be the imbalanced medical conditions in different regions of China. As Kashi is located in the northwest of China, it lags behind other Chinese eastern regions in terms of economy and medical resources. Worse, the first outbreak and second wave of COVID-19 pandemic burdened the medical resources situation in Kashi. The shortage and impaired medical resources also aroused medical staffs' worry and fear, which may account for the higher prevalence of mental health problems among them.

In this study, we identified that minority ethnicities were more likely to have anxiety symptoms. A prior study done by Wang also revealed that the Tibetan (minority) cancer inpatients had a significantly higher incidence of anxiety than that of the Han (majority) cancer inpatients (25). The differences in culture and religious beliefs are thought to be the reason (25). Meanwhile, another study compares the death anxiety between Han and Tibetan ethnic group, showing that Tibetan respondents express more death anxiety, fear of death, death avoidance, and escape acceptance than the Han participants (26). Such differences are considered to attribute to their different culture, religious beliefs, and even implicit attitudes. On the contrary, a higher education level was found as a protective factor for insomnia and anxiety symptoms, which is consistent with previous studies (27, 28). Compared with those with a higher degree, individuals with a low education level may have more difficulty in understanding and confronting the pandemic, which may lead to the fear of COVID-19. Such fear may then particularly have an impact on the mental health of medical staffs with a low education level.

Worrying about infection was also identified as another factor associated with depressive symptoms while spending more than 30 min on pandemic-related information before sleep was also associated with acute stress symptoms. Some previous studies have discovered the association between worry and depression, which is consistent with our finding (29, 30). In regards to the relationship between time spent on pandemic information and acute stress symptoms, the association has not been reported yet, despite a previous study reveals bidirectional associations between the duration of mobile phone use and various sleep and mental outcomes such as depression and anxiety (31). Several mechanisms may explain the association. On one hand, the myriad of information received may increase their cognitive or emotional burden and increase their vulnerability to depression and anxiety (32), which may result in their vulnerability to acute stress. On the other hand, while the time spent on pandemic information may reflect the worry about the pandemic, stress reactivity is exacerbated by daily pandemic worry (33). However, further study should be completed to confirm this association.

We also found that the medical staffs who scored higher in the neuroticism subscale of CBF-PI-15 were more likely to develop depression, anxiety, acute stress, and insomnia symptoms, while those who scored higher in the extraversion subscale were less likely to suffer acute stress symptoms. Neuroticism consists of a person's tendency to experience negative feelings, anxiety, and psychological distress (34), while extraversion refers to the inclination to be energetic, sociable, and assertive, and conscientiousness encompasses organization, self-discipline, and determination (35). Several previous studies reveal that neuroticism is positively associated with various psychological problems, which is consistent with our study (36–39). At the same time, higher levels of extraversion are also found to be related to positive health outcomes (40).

Medical staffs who experienced more anxiety symptoms were found to have poorer social support. The same result comes from a cross-cultural study, in which resilience and social support are universal interrelated protective factors for mental health (41). Social support, which has been defined as information from others that one is loved and cared for, esteemed and valued, and part of a network of communication and mutual obligations (42), is widely recognized to have a great impact on people's health. It can be distinguished into structural and functional measures, and further measures can be divided into emotional, instrumental, and informational support (43). Facing the pandemic, medical staffs are in extreme need of functional measures of support, especially the emotional ones and the informational ones. Thus, obtaining better social support would help medical staffs reduce their risk of suffering from mental health problems.

In summary, continuous psychological support would be particularly important to medical staffs as the pandemic remains prolonged. Special care should be paid to those of minority ethnicities and those not well-educated. Besides, identifying the medical staffs who are more neurotic and giving them more special care may help to reduce their risk of experiencing psychological distress. Helping medical staffs to handle their worry and fear, to maintain good interpersonal relationship, and to have access to necessary functional support also plays an important role in the avoidance of negative health conditions.

To our best knowledge, this is the first study conducted to systematically investigate mental health conditions and to explore the related social psychological factors among medical staffs in Xinjiang who experienced the second wave of the COVID-19 pandemic. Our findings help to fill the gap in the understanding of the mental health status of medical staffs in Xinjiang during the resurgence period.

Our study has several limitations. First, due to the relatively poor economic resources as well as great language differences in Xinjiang, the study could hardly be conducted in a wider range. Because of such inconveniences, the snowball sampling method was used and the sample size was small in this survey, leaving the coverage and representativeness of our study limited. Second, this was a cross-sectional study, which means the associations between mental health conditions and predictors could not be considered as causal relationships. Third, all symptoms in the survey were self-reported instead of being diagnosed by profession, which may lead to report bias. Fourth, only medical staffs were included in this study, thus their mental health problems could not be compared directly with the public during this second wave of the pandemic, which requires further investigation. At last, other potential predictors, such as marital status or history of physical illness, also need special attention. However, this study only focused on some basic demographic factors and psychological factors, and thus did not elaborate on other possible related factors, which warrants further research.

## Conclusion

In our study, the prevalence rate of depressive, anxiety, acute stress, and insomnia symptoms was high among medical staffs in Kashi, Xinjiang who experienced the second wave of the COVID-19 pandemic. Several factors were found to be associated with mental health conditions. These findings could help identify medical staffs at risk for mental health problems and may help make precise mental health intervention policies during the resurgence period. Our study may also call for further research into Xinjiang during the COVID-19 pandemic.

## Data Availability Statement

The raw data supporting the conclusions of this article will be made available by the authors, without undue reservation.

## Ethics Statement

The studies involving human participants were reviewed and approved by The Ethics Committee of Nanfang Hospital, Southern Medical University. The patients/participants provided their written informed consent to participate in this study.

## Author Contributions

YZ and JG shared the first authorship. BZ, MA, and YZ conceived and designed this study. YZ and JG conceived and conducted statistical analyses, with additional advice regarding analyses contributed by SL, QZ, BZ, RA, AK, AS, YX, and AT. JG drafted the manuscript, and all authors contributed to editing it and approved the final manuscript.

## Conflict of Interest

The authors declare that the research was conducted in the absence of any commercial or financial relationships that could be construed as a potential conflict of interest.

## References

[1] The Government of Xinjiang Uygur Autonomous Region of China. Two novel coronavirus cases were confirmed for the first time in Xinjiang. (2020). Avaialable online at: http://www.xinjiang.gov.cn/xinjiang/c100225/202001/387cab88b6264cc5995d045f9d5d601a.shtml (accessed 28 December, 2020).

[2] The Government of Xinjiang Uygur Autonomous Region of China. No new confirmed cases or asymptomatic infections were reported in Xinjiang (including the XPCC) on August 18. (2020). Avaialable online at: http://www.xinjiang.gov.cn/xinjiang/tzgg/202008/55245d1e0cf24bedb7cd3129f35f175b.shtml (accessed 23 March, 2021).

[3] National Health Commission of the PRC. August 21 Daily briefing on novel coronavirus cases in China. (2020). Avaialable online at: http://www.nhc.gov.cn/xcs/yqtb/202008/d3ac3a68c25249adb5df9f0cb58820f3.shtml (accessed March 23, 2021).

[4] The Government of Xinjiang Uygur Autonomous Region of China. On October 24, there was no new confirmed case in Xinjiang (including XPCC). (2020). Avaialable online at: http://www.xinjiang.gov.cn/xinjiang/tzgg/202010/59429f3d9eaa4f09bf678dbc1bb8a57f.shtml (accessed 28 December, 2020).

[5] Wu T Jia X Shi H Niu J Yin X Xie J. Prevalence of mental health problems during the COVID-19 pandemic: a systematic review and meta-analysis. J Affect Disord. (2020) 281:91–8. 10.1016/j.jad.2020.11.117 33310451 PMC7710473

[6] Liu CY Yang YZ Zhang XM Xu X Dou QL Zhang WW. The prevalence and influencing factors in anxiety in medical workers fighting COVID-19 in China: a cross-sectional survey. Epidemiol Infect. (2020) 148:e98. 10.1017/S0950268820001107 32430088 PMC7251286

[7] Zhang C Yang L Liu S Ma S Wang Y Cai Z. Survey of insomnia and related social psychological factors among medical staff involved in the 2019 novel coronavirus disease outbreak. Front Psychiatry. (2020) 11:3542175. 10.2139/ssrn.3542175 32346373 PMC7171048

[8] Zhu J Sun L Zhang L Wang H Fan A Yang B. Prevalence and influencing factors of anxiety and depression symptoms in the first-line medical staff fighting against COVID-19 in Gansu. Front Psychiatry. (2020) 11:3550054. 10.2139/ssrn.3550054 32411034 PMC7202136

[9] Wang Y Duan Z Peng K Li D Ou J Wilson A. Acute stress disorder among frontline health professionals during the COVID-19 outbreak: a structural equation modelling investigation. Psychosom Med. (2020). 10.1097/PSY.0000000000000851. [Epub ahead of print]. 32815855

[10] Xiao H Zhang Y Kong D Li S Yang N. The effects of social support on sleep quality of medical staff treating patients with Coronavirus Disease 2019 (COVID-19) in january and february 2020 in China. Med Sci Monit. (2020) 26:e923549. 10.12659/MSM.923549 32132521 PMC7075079

[11] Nikčević AV Marino C Kolubinski DC Leach D Spada MM. Modelling the contribution of the Big Five personality traits, health anxiety, and COVID-19 psychological distress to generalised anxiety and depressive symptoms during the COVID-19 pandemic. J Affect Disord. (2021) 279:578–84. 10.1016/j.jad.2020.10.053 33152562 PMC7598311

[12] Chang J Hu L Guan Q. Investigation and analysis of psychological stress response of medical staff in a grade II hospital of xinjiang army during the outbreak of coronavirus disease. J Nongken Med. (2020) 42:328–32. Available online at: https://kns.cnki.net/kcms/detail/detail.aspx?FileName=NKYX202004011&DbName=CJFQ2020

[13] Kroenke K Spitzer RL Williams JB. The PHQ-9: validity of a brief depression severity measure. J Gen Intern Med. (2001) 16:606–13. 10.1046/j.1525-1497.2001.016009606.x 11556941 PMC1495268

[14] Lowe B Decker O Muller S Brahler E Schellberg D Herzog W. Validation and standardization of the Generalized Anxiety Disorder Screener (GAD-7) in the general population. Med Care. (2008) 46:266–74. 10.1097/MLR.0b013e318160d093 18388841

[15] Thoresen S Tambs K Hussain A Heir T Johansen VA Bisson JI. Brief measure of posttraumatic stress reactions: impact of Event Scale-6. Soc Psych Psych Epid. (2010) 45:405–12. 10.1007/s00127-009-0073-x 19479171

[16] Li X Lv S Liu L Chen R Chen J Liang S. COVID-19 in Guangdong: immediate perceptions and psychological impact on 304,167 college students. Front Psychol. (2020) 11:2024. 10.3389/fpsyg.2020.02024 32903445 PMC7434974

[17] Morin CM Belleville G Belanger L Ivers H. The Insomnia Severity Index: psychometric indicators to detect insomnia cases and evaluate treatment response. Sleep. (2011) 34:601–8. 10.1093/sleep/34.5.601 21532953 PMC3079939

[18] Zhang X Wang MC He L Jie L Deng J. The development and psychometric evaluation of the Chinese Big Five Personality Inventory-15. PLoS ONE. (2019) 14:e0221621. 10.1371/journal.pone.0221621 31454383 PMC6771307

[19] Jiang Q Zhu Y. Further explorations for a coping style questionnaire. Chin J Behav Med Sci. (1999) 8:167–9.

[20] Zimet GD Dahlem NW Zimet SG Farley GK. (1988). The multidimensional scale of perceived social support. J Person Assess 52:30–41. 10.1207/s15327752jpa5201_2

[21] Pappa S Ntella V Giannakas T Giannakoulis VG Papoutsi E Katsaounou P. Prevalence of depression, anxiety, and insomnia among healthcare workers during the COVID-19 pandemic: a systematic review and meta-analysis. Brain Behav Immun. (2020) 88:901–7. 10.1016/j.bbi.2020.05.026 32437915 PMC7206431

[22] Taha S Matheson K Cronin T Anisman H. Intolerance of uncertainty, appraisals, coping, and anxiety: the case of the 2009 H1N1 pandemic. Br J Health Psychol. (2014) 19:592–605. 10.1111/bjhp.12058 23834735

[23] Bakioglu F Korkmaz O Ercan H. Fear of COVID-19 and positivity: mediating role of intolerance of uncertainty, depression, anxiety, and stress. Int J Ment Health Addict. (2020) 28:1–14. 10.1007/s11469-020-00331-y 32837421 PMC7255700

[24] Lauriola M Carleton RN Tempesta D Calanna P Socci V Mosca O. A correlational analysis of the relationships among intolerance of uncertainty, anxiety sensitivity, subjective sleep quality, insomnia symptoms. Int J Environ Res Public Health. (2019) 16:3253. 10.3390/ijerph16183253 31491841 PMC6765836

[25] Wang Y Mei C Fu Y Yue Z Jiang Y Zhu J. Anxiety and depression among Tibetan inpatients with cancer: a multicenter investigation. Ann Palliat Med. (2020) 9:3776–84. 10.21037/apm-20-1721 33222453

[26] Yin F He Y He Y Shen H Ip K. A comparative study of death anxiety and death attitudes in Han and Tibetan ethnic groups. Death Stud. (2020). 10.1080/07481187.2020.1802791. [Epub ahead of print]. 32780652

[27] Xiang YT Ma X Cai ZJ Li SR Xiang YQ Guo HL. The prevalence of insomnia, its sociodemographic and clinical correlates, and treatment in rural and urban regions of Beijing, China: a general population-based survey. Sleep. (2008) 31:1655–62. 10.1093/sleep/31.12.1655 19090321 PMC2603488

[28] Shi L Lu Z Que J Huang X Liu L Ran M. Prevalence of and risk factors associated with mental health symptoms among the general population in china during the Coronavirus Disease 2019 pandemic. JAMA Netw Open. (2020) 3:e2014053. 10.1001/jamanetworkopen.2020.14053 32609353 PMC7330717

[29] Yook K Kim KH Suh SY Lee KS. Intolerance of uncertainty, worry, and rumination in major depressive disorder and generalized anxiety disorder. J Anxiety Disord. (2010) 24:623–8. 10.1016/j.janxdis.2010.04.003 20439149

[30] Gorday JY Rogers ML Joiner TE. Examining characteristics of worry in relation to depression, anxiety, and suicidal ideation and attempts. J Psychiatr Res. (2018) 107:97–103. 10.1016/j.jpsychires.2018.10.004 30384092

[31] Liu S Wing YK Hao Y Li W Zhang J Zhang B. The associations of long-time mobile phone use with sleep disturbances and mental distress in technical college students: a prospective cohort study. Sleep. (2019) 42:213. 10.1093/sleep/zsy213 30395300

[32] Yen CF Tang TC Yen JY Lin HC Huang CF Liu SC. Symptoms of problematic cellular phone use, functional impairment and its association with depression among adolescents in Southern Taiwan. J Adolesc. (2009) 32:863–73. 10.1016/j.adolescence.2008.10.006 19027941

[33] Nelson NA Bergeman CS. Daily stress processes in a pandemic: the effects of worry, age, and affect. Gerontologist. (2020) 61:196–204. 10.1093/geront/gnaa187 33186445 PMC7717331

[34] Maggio MG Cuzzola MF Latella D Impellizzeri F Todaro A Rao G. How personality traits affect functional outcomes in patients with multiple sclerosis: a scoping review on a poorly understood topic. Mult Scler Relat Disord. (2020) 46:102560. 10.1016/j.msard.2020.102560 33049463

[35] Bibbey A Carroll D Roseboom TJ Phillips AC de Rooij. Personality SR, and physiological reactions to acute psychological stress. Int J Psychophysiol. (2013) 90:28–36. 10.1016/j.ijpsycho.2012.10.018 23147393

[36] Kotov R Gamez W Schmidt F Watson D. Linking “big” personality traits to anxiety, depressive, and substance use disorders: a meta-analysis. Psychol. Bull. (2010) 136:768–821. 10.1037/a0020327 20804236

[37] Duggan KA Friedman HS McDevitt EA Mednick SC. Personality and healthy sleep: the importance of conscientiousness and neuroticism. PLoS ONE. (2014) 9:e90628. 10.1371/journal.pone.0090628 24651274 PMC3961248

[38] Kennair L Solem S Hagen R Havnen A Nysaeter TE Hjemdal O. Change in personality traits and facets (NEO-PI-R) following metacognitive therapy or cognitive behavior therapy for generalized anxiety disorder: results from a randomized controlled trial. Clin Psychol Psychother. (2020). 10.1002/cpp.2541. [Epub ahead of print]. 33338315

[39] Robillard R Saad M Edwards J Solomonova E Pennestri M Daros A. Social, financial and psychological stress during an emerging pandemic: observations from a population survey in the acute phase of COVID-19. BMJ Open. (2020) 10:e043805. 10.1136/bmjopen-2020-043805 33310814 PMC7735085

[40] Macía P Gorbeña S Gómez A Barranco M Iraurgi I. Role of neuroticism and extraversion in the emotional health of people with cancer. Heliyon. (2020) 6:e04281. 10.1016/j.heliyon.2020.e04281 32671245 PMC7339056

[41] Brailovskaia J Schönfeld P Zhang XC Bieda A Kochetkov Y Margraf J. A cross-cultural study in germany, russia, and china: are resilient and social supported students protected against depression, anxiety, and stress? Psychol Rep. (2017) 121:265–81. 10.1177/0033294117727745 28836915

[42] Kim HS Sherman DK Taylor SE. Culture and social support. Am Psychol. (2008) 63:518–26. 10.1037/0003-066X 18793039

[43] Helgeson VS. Social support and quality of life. Qual Life Res. (2003) 12:25–31. 10.1023/A:1023509117524 12803308

